# The effect of foot landing position on biomechanical risk factors associated with anterior cruciate ligament injury

**DOI:** 10.1186/s40634-016-0049-1

**Published:** 2016-06-18

**Authors:** Andrew A. Tran, Corey Gatewood, Alex H. S. Harris, Julie A. Thompson, Jason L. Dragoo

**Affiliations:** Stanford University School of Medicine, Stanford University, Stanford, CA USA; Department of Orthopaedic Surgery, Stanford University, Stanford, CA USA; Department of Bioengineering, Stanford University, Stanford, CA USA; Human Performance Lab, Stanford Sports Medicine Center, 341 Galvez Street, Stanford, CA 94305 USA

**Keywords:** Anterior cruciate ligament, ACL, Landing pattern, Biomechanics, Dynamic valgus

## Abstract

**Background:**

Identification of biomechanical risk factors associated with anterior cruciate ligament (ACL) injury can facilitate injury prevention. The purpose of this study is to investigate the effects of three foot landing positions, “toe-in”, “toe-out” and “neutral”, on biomechanical risk factors for ACL injury in males and females. The authors hypothesize that 1) relative to neutral, the toe-in position increases the biomechanical risk factors for ACL injury, 2) the toe-out position decreases these biomechanical risk factors, and 3) compared to males, females demonstrate greater changes in lower extremity biomechanics with changes in foot landing position.

**Methods:**

Motion capture data on ten male and ten female volunteers aged 20–30 years (26.4 ± 2.50) were collected during double-leg jump landing activities. Subjects were asked to land on force plates and target one of three pre-templated foot landing positions: 0° (“neutral”), 30° internal rotation (“toe-in”), and 30° external rotation (“toe-out”) along the axis of the anatomical sagittal plane. A mixed-effects ANOVA and pairwise Tukey post-hoc comparison were used to detect differences in kinematic and kinetic variables associated with biomechanical risk factors of ACL injury between the three foot landing positions.

**Results:**

Relative to neutral, landing in the toe-in position increased peak hip adduction, knee internal rotation angles and moments (*p* < 0.01), and peak knee abduction angle (*p* < 0.001). Landing in the toe-in position also decreased peak hip flexion angle (*p* < 0.001) and knee flexion angle (*p* = 0.023). Landing in the toe-out position decreased peak hip adduction, knee abduction, and knee internal rotation angles (all *p* < 0.001). Male sex was associated with a smaller increase in hip adduction moment (*p* = 0.043) and knee internal rotation moment (*p* = 0.032) with toe-in landing position compared with female sex.

**Conclusions:**

Toe-in landing position exacerbates biomechanical risk factors associated with ACL injury, while toe-out landing position decreases these factors.

## Background

Current evidence suggests that anterior cruciate ligament (ACL) injury risk is multifactorial (Hewett et al. 2006), involving anatomical, hormonal, neuromuscular, and biomechanical factors (Alentorn-Geli et al. 2009; Boden et al. 2000; Feagin and Lambert 1985; Hewett et al. 2000; Hewett et al. 2006; Shultz and Schmitz 2012). Identifying modifiable biomechanical risk factors can facilitate ACL injury prevention. A combination of increased hip adduction and internal rotation, decreased knee flexion, increased knee abduction and internal or external tibial rotation, termed “dynamic knee valgus”, may increase the risk of ACL injury (Boden et al. 2009; Hewett et al. 2009; Hewett et al. 2006; Schmitz et al. 2008).

Foot landing positions, also termed “toe-in” and “toe-out”, can affect lower extremity biomechanics. For example, toe-in landing has previously been associated with increased tibial rotation and knee valgus in video analyses of handball players (Olsen et al. 2004). Padua et al. included toe-in or toe-out of greater than 30° as a “high-risk” position in the Landing Error Scoring System (LESS), a validated tool to screen for potential high-risk movement patterns during jump-landing tasks (Padua et al. 2009). However, few studies have attempted to quantify the effect of high-risk foot landing positions on lower extremity movement patterns. A better understanding of this effect can help guide movement pattern modification for specific injury risk factors associated with ACL injury.

Biomechanical risk factors associated with ACL injury are amplified in female athletes, who are at a 2-9 times greater risk of ACL injury than their male counterparts (Prodromos et al. 2007; Toth and Cordasco 2001). Anatomic and hormonal risk factors aside, compared to males, females demonstrate greater landing forces and greater knee frontal plane motion during cutting and jump-landing (Ford et al. 2005; Ford et al. 2003; Quatman et al. 2006; Shultz et al. 2007). Compared to males, female athletes have been found to demonstrate decreased hip and knee flexion, increased knee valgus, increased knee rotation, and increased ankle eversion during jump-landing activities (Chappell et al. 2007; Ford et al. 2005; Malinzak et al. 2001; McLean et al. 2005). However, the role of foot landing position on these differences has not been studied.

The purpose of this study was to investigate the effect of foot landing position on biomechanical risk factors for ACL injury in males and females. We hypothesized that 1) compared to landing at neutral foot position (0° rotation), landing in the toe-in position would increase lower extremity biomechanical risk factors associated with ACL injury, 2) landing in the toe-out position would mitigate these factors and 3) females would display greater changes in these factors when changing foot landing position compared to males.

## Methods

Institutional review board approval was obtained and written informed consent was collected from ten male and ten female volunteers between the ages of 20 and 30 (Table 1). Subjects were excluded from this study if they had a self-reported functional impairment, any history of lower extremity surgery, a current lower extremity injury, or a history of ACL injury.

**Table 1:** Mean ± SD subject demographics

	Total (*n* = 20)	Male (*n* = 10)	Female (*n* = 10)	*p*
Age	26.4 ± 2.50	26.1 ± 1.45	26.7 ± 3.30	0.304
Height (m)	1.70 ± 0.08	1.75 ± 0.05	1.65 ± 0.06	< 0.001*
Weight (kg)	67.1 ± 14.3	76.9 ± 13.9	57.3 ± 5.24	< 0.001*

*p*-value represents t-test probability comparing male and female subgroups. *denotes statistical significance at α = 0.05

### Lab testing

**Fig. 1:**
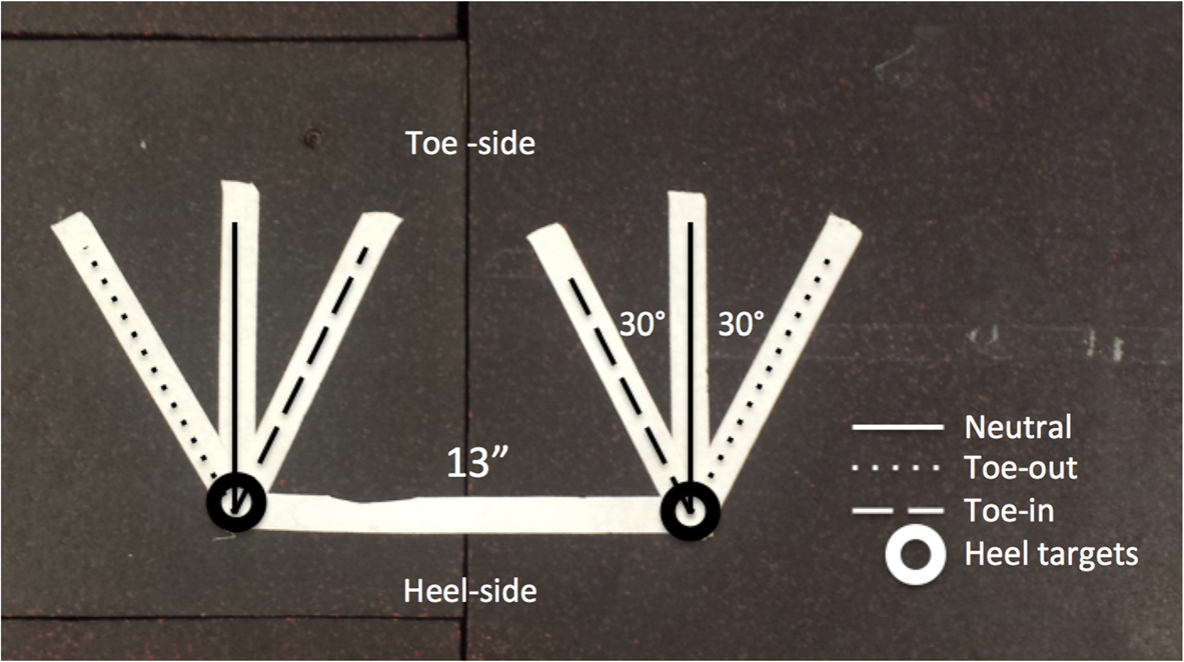
Foot landing position template. Subjects were instructed to land at neutral, toe-out, or toe-in landing position

**Fig. 2:**
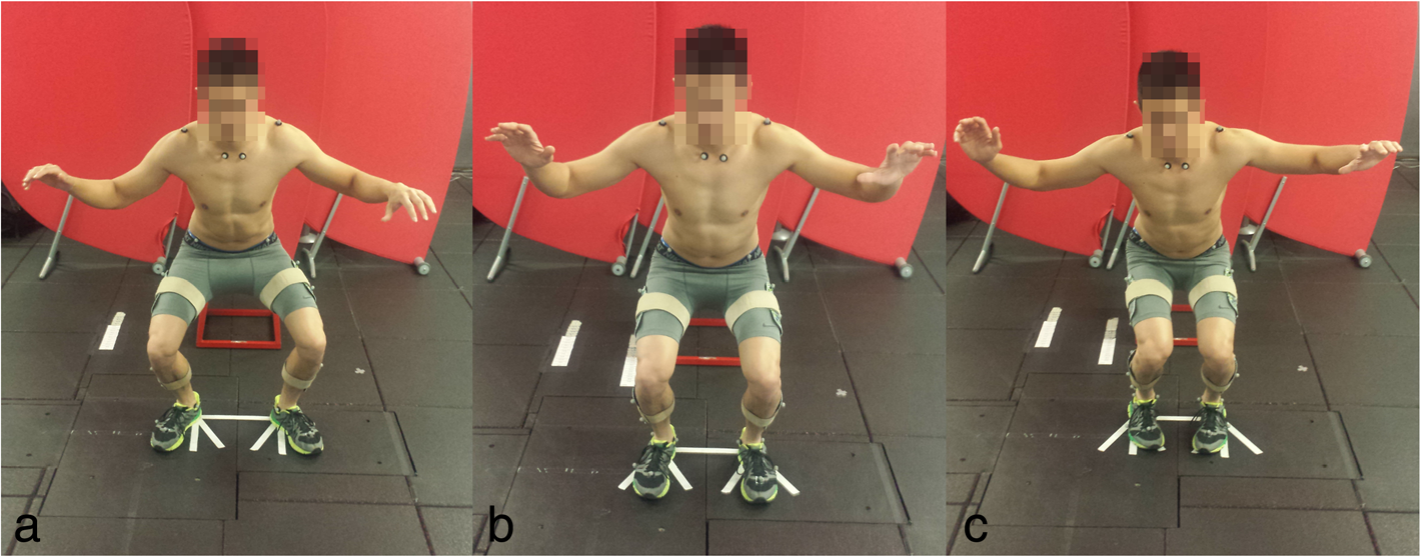
Jump-landing activity for **a**) toe-out **b**) neutral and **c**) toe-in landing positions

The positions of 36 retro-reflective markers were recorded at 200Hz using an 8-camera optical motion capture system (Motion Analysis Corp., Santa Rosa, CA, USA) and were synchronized with measurements from three floor-mounted force plates collected at 2000Hz (Bertec Corporation, Columbus, OH, USA). Each subject performed an initial static standing calibration trial. Subjects then completed 3 trials each of 3 jump-landing tasks from a 30-cm box, landing on one of three different foot position templates marked with tape on the force plates (Fig. 1). The templates were standardized for all subjects and positioned such that each foot would land on a separate force plate at a 13-in. stance width. Neutral foot position was defined as zero degrees rotation from a line directed anteriorly along the sagittal plane of the body. Toe-in and toe-out landing position were defined as 30° internal and external rotation, respectively, relative to the neutral position and using the back of the heel as the pivot reference. The jump sequence was randomized using a random number generator and subjects were instructed to land on either the neutral, toe-in, or toe-out foot position template. Subjects jumped forward from the box onto the appropriate template at a distance of 50 % body height, and immediately performed a countermovement jump to achieve maximal height (Fig. 2). Subjects received no prior instruction on landing mechanics, with the exception of being told to target the appropriate foot position template.

### Musculoskeletal modeling

The ground reaction force data were low-passed filtered using a fourth-order critically damped filter with a cutoff frequency of 30Hz. We analyzed trials for each activity and subject using OpenSim software version 3.2 (Delp et al. 2007). A generic 34°-of-freedom musculoskeletal model was scaled to match the anthropometry of the individual subjects using markers located on anatomical landmarks (Caruthers et al. 2016). Joint angles were estimated using the inverse kinematics tool, which reproduced the experimental gait patterns in the scaled model using a weighted least-squares approach to minimize the differences between the experimental marker locations and the model’s virtual marker locations. Kinematics were then filtered at 30Hz using OpenSim (Low-pass IIR Butterworth, 3^rd^ order) and input to the inverse dynamics tool to estimate joint moments. The same 30Hz filter used for the ground reaction force data was then re-applied to the joint moments in order to reduce kinetic artifacts (Bisseling and Hof 2006).

### Statistical analysis

For each trial, we determined values at initial contact (IC) and peak values during weight acceptance, defined as the interval between initial contact and peak knee flexion. Joint moments were normalized by subject bodyweight and height. All joint moments are reported as external moments. A mixed-effects ANOVA, to account for the nesting of jumps within subject within position, was used to estimate differences in kinetics and kinematics between foot landing positions. Pairwise differences between neutral foot position and external or internal landing positions were estimated from the models using Tukey post-hoc comparisons and a 95 % family-wise confidence level. To examine if the effect of foot position differed between males and females, a sex-by-foot position interaction was entered into the models. An alpha less than 0.05 was considered significant. Statistical analysis was performed using R software (R Core Team 2015).

## Results

### Kinematics

Compared to neutral foot landing position, toe-in was associated with increased hip adduction angle (IC, peak), knee abduction angle (IC, peak), knee internal rotation angle (IC, peak), and increased ankle dorsiflexion angle (peak). Toe-in was associated with decreased hip flexion angle (peak), knee flexion angle (peak), knee external rotation angle (peak), and foot pronation angle (IC, peak). Toe-out was associated with increased hip abduction angle (IC, peak) and increased foot pronation angle (IC). Toe-out was associated with decreased knee abduction angle (IC, peak), knee external rotation angle (IC), and ankle dorsiflexion (peak) (Table 2).

**Table 2:** Summary of kinematic data at initial contact (IC) and peak during weight acceptance (peak) for all subjects

		IC		Peak	
Variable	Foot Position	Mean (°) ± SD	*p*	Mean (°) ± SD	*p*
Trunk Flexion Angle	Neutral	22.58 ± 7.08		35.26 ± 11.87	
	Toe-out	21.87 ± 7.10	0.544	34.25 ± 11.04	0.506
	Toe-in	22.21 ± 7.70	0.864	35.63 ± 14.10	1.00
Trunk Lateral Sway Angle	Neutral	0.52 ± 3.00		2.19 ± 3.01	
	Toe-out	0.33 ± 2.71	0.900	2.44 ± 2.72	0.830
	Toe-in	0.68 ± 3.95	0.990	2.68 ± 3.70	0.703
Hip Flexion Angle	Neutral	36.48 ± 6.52		74.24 ± 17.06	
	Toe-out	35.84 ± 7.18	0.941	74.93 ± 17.53	0.162
	Toe-in	36.48 ± 7.57	0.581	70.26 ± 19.45	< 0.001*
Hip Adduction Angle	Neutral	-7.72 ± 3.87		-3.73 ± 3.02	
	Toe-out	-14.72 ± 3.77	< 0.001*	-13.69 ± 3.40	< 0.001*
	Toe-in	-1.86 ± 4.66	< 0.001*	3.46 ± 3.89	< 0.001*
Knee Flexion Angle	Neutral	26.55 ± 5.23		90.44 ± 14.71	
	Toe-out	27.51 ± 5.57	0.362	92.69 ± 15.02	0.088
	Toe-in	26.56 ± 5.90	0.986	88.22 ± 16.00	0.023*
Knee Abduction Angle	Neutral	-3.62 ± 3.80		7.68 ± 7.54	
	Toe-out	-6.09 ± 5.65	< 0.001*	4.84 ± 9.06	< 0.001*
	Toe-in	0.84 ± 5.96	< 0.001*	10.00 ± 8.43	< 0.001*
Knee Internal Rotation Angle	Neutral	3.78 ± 6.35		15.11 ± 6.29	
	Toe-out	2.69 ± 7.48	< 0.001*	14.49 ± 7.55	< 0.001*
	Toe-in	11.94 ± 5.24	< 0.001*	18.08 ± 4.59	< 0.001*
Knee External Rotation Angle	Neutral			-3.03 ± 4.22	
	Toe-out			3.08 ± 5.01	0.271
	Toe-in			-10.07 ± 1.54	< 0.001*
Ankle Dorsiflexion Angle	Neutral	-18.25 ± 13.35		20.95 ± 4.26	
	Toe-out	-19.10 ± 13.74	0.852	18.43 ± 5.18	< 0.001*
	Toe-in	-17.56 ± 12.06	0.977	23.87 ± 4.52	< 0.001*
Foot Pronation Angle	Neutral	1.90 ± 5.04		12.53 ± 3.76	
	Toe-out	2.78 ± 6.91	0.026*	13.78 ± 3.52	0.108
	Toe-in	-5.52 ± 6.64	< 0.001*	1.36 ± 6.83	< 0.001*

*p*-values represent Tukey post-hoc comparisons to neutral foot landing position (95 % family-wise confidence level). *denotes statistical significance

### Kinetics

Compared to neutral foot landing position, toe-in was associated with increased hip adduction moment (IC, peak), knee flexion moment (peak), and knee internal rotation moment (peak). Toe-in was associated with decreased knee external rotation moment (peak) and decreased foot pronation moment (IC, peak). Toe-out was associated with increased trunk flexion moment (peak), knee internal rotation moment (peak), and foot pronation moment (IC, peak) (Table 3).

**Table 3:** Summary of kinetic data at initial contact (IC) and peak during weight acceptance (peak) for all subjects. Values are normalized to % body weight × height

		IC		Peak	
Variable	Foot Position	Mean ± SD	*p*	Mean ± SD	*p*
Trunk Flexion Moment	Neutral	-1.36 ± 3.60		7.37 ± 3.20	
	Toe-out	-1.36 ± 2.52	1.00	6.57 ± 3.14	0.048*
	Toe-in	-1.34 ± 2.31	0.987	6.87 ± 2.87	0.701
Trunk Lateral Moment	Neutral	0.70 ± 6.56		5.49 ± 3.20	
	Toe-out	0.05 ± 5.02	0.698	5.58 ± 3.89	0.995
	Toe-in	0.12 ± 3.07	0.815	9.64 ± 5.29	0.092
Hip Flexion Moment	Neutral	1.12 ± 8.60		13.70 ± 5.73	
	Toe-out	-0.22 ± 7.40	0.917	14.04 ± 5.30	0.620
	Toe-in	2.67 ± 10.42	0.948	13.18 ± 4.40	0.767
Hip Adduction Moment	Neutral	-1.66 ± 3.57		3.73 ± 3.12	
	Toe-out	-1.25 ± 4.58	0.374	2.58 ± 1.87	0.745
	Toe-in	0.42 ± 4.40	0.043*	5.26 ± 4.36	0.001*
Knee Flexion Moment	Neutral	0.89 ± 3.81		13.95 ± 5.80	
	Toe-out	1.72 ± 4.15	0.447	14.89 ± 8.27	0.259
	Toe-in	0.46 ± 5.30	0.129	15.53 ± 8.21	0.0074*
Knee Abduction Moment	Neutral	0.85 ± 1.72		4.85 ± 2.76	
	Toe-out	0.74 ± 2.10	0.935	5.19 ± 3.38	0.814
	Toe-in	1.31 ± 2.71	0.336	5.45 ± 4.12	0.314
Knee Internal Rotation Moment	Neutral			0.94 ± 0.60	
	Toe-out			1.05 ± 1.13	< 0.001*
	Toe-in			1.22 ± 1.02	< 0.001*
Knee External Rotation Moment	Neutral	0.18 ± 0.43		0.43 ± 0.34	
	Toe-out	0.18 ± 0.41	0.605	0.49 ± 0.32	0.519
	Toe-in	0.11 ± 0.47	0.628	0.15 ± 0.44	< 0.001*
Ankle Dorsiflexion Moment	Neutral	1.64 ± 1.98		7.68 ± 2.93	
	Toe-out	1.72 ± 1.79	0.929	8.23 ± 3.58	0.784
	Toe-in	1.57 ± 1.91	0.925	8.68 ± 3.94	0.157
Foot Pronation Moment	Neutral	1.31 ± 1.16		3.49 ± 1.37	
	Toe-out	1.55 ± 1.03	< 0.001*	3.88 ± 1.57	0.026*
	Toe-in	0.87 ± 0.86	< 0.001*	2.17 ± 1.00	< 0.001*

*p*-values represent Tukey post-hoc comparisons to neutral foot landing position (95 % family-wise confidence level). *denotes statistical significance

### Sex differences

**Fig. 3:**
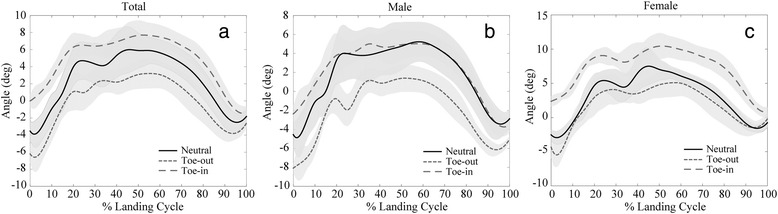
Average knee abduction angle throughout the landing cycle for neutral, toe-out, and toe-in foot landing position for **a**) males and females combined, **b**) males only and **c**) females only. The landing cycle is defined as the time from initial contact to toe off

Differences in kinematic and kinetic variables relative to neutral foot landing position were compared between male and female sex. Toe-out landing position in males was associated with a greater increase in hip abduction angle (IC: Δ = 1.67 ± 0.789°, *p* = 0.036; peak: Δ = 2.45 ± 0.868°, *p* = 0.005) compared to females. Toe-in landing position in males was associated with a smaller increase in knee internal rotation moment (peak: Δ = 0.373 ± 0.172, *p* = 0.032), greater reduction in foot pronation moment (peak: Δ = 0.750 ± 0.280, *p* = 0.008), smaller increase in knee abduction angle (IC: Δ = -2.93 ± 0.804°, *p* < 0.001) (Fig. 3), and greater reduction in foot pronation angle (peak: Δ = 2.75 ± 1.24°, *p* = 1.028) compared to females.

Of note, there were several trends towards significance. Compared to females, toe-out landing position in males trended towards a greater increase in knee flexion moment at initial contact (Δ = 2.52 ± 1.37, *p* = 0.068), a greater reduction in peak knee abduction moment (Δ = 1.09 ± 0.631, *p* = 0.085), and a greater reduction in knee abduction angle at initial contact (Δ = 1.42 ± 0.792°, *p* = 0.075). Toe-in landing position in males trended towards a smaller increase in peak knee abduction angle (Δ = -2.34 ± 1.25°, *p* = 0.062) compared to females.

## Discussion

Foot landing position serves as an observable and recordable movement pattern and is therefore a prime target for movement pattern modification. Our results demonstrate that foot landing position can affect multiple lower extremity biomechanical factors associated with ACL injury.

Excessive knee abduction during cutting and landing activities increases ACL injury risk (Hewett et al. 1999). We found that landing in a toe-in position increases knee abduction angle. Furthermore, landing in a toe-in position increases hip adduction angle and moment, which are associated with increased risk of ACL injury (Boden et al. 2009; Hewett et al. 2009). Landing at toe-in was also found to increase tibial internal rotation angle and moment. Biomechanical and MRI studies have shown that greater internal tibial rotation increases ACL load (Fung et al. 2007; Fung and Zhang 2003; Markolf et al. 1995). Furthermore, tibial internal rotation combined with knee valgus was found to increase ACL strain more than either individually (Shin et al. 2011). These findings suggest that an excessive toe-in landing position should be avoided and may be a target for movement pattern modification.

Toe-out landing position was found to be associated with decreased knee abduction angles and increased tibial external rotation angles. While excessive external tibial rotation may increase impingement of the ACL against the intercondylar notch, only minimal increases in ACL strain have been observed with isolated tibial external rotation (Markolf et al. 1995). While toe-out position does not appear to be detrimental in jump landing tasks, analysis has shown that tibial external rotation in conjunction with knee valgus is a common movement pattern observed in non-contact ACL injury (Koga et al. 2010). Future study on change of direction movements such as cutting may be warranted to determine the effect of the toe-out position on ACL injury risk.

Male sex appears to be somewhat protective against the increase in lower extremity biomechanical factors associated with toe-in landing position. Females have been shown to exhibit more risky lower extremity biomechanics associated with ACL injury compared with males (Fagenbaum and Darling 2003; Ford et al. 2005; Ford et al. 2003; Quatman et al. 2006; Shultz et al. 2007). Relative to their neutral foot position, females displayed greater increases in risky movement patterns with toe-in landing compared to males, suggesting that changes in foot landing position may have greater effects on lower extremity biomechanics in females.

This study has several limitations. Evaluating a few pre-determined landing patterns in a lab differs from the large number of possible landing positions during dynamic activities. Additionally, we evaluated a symmetric jump-landing task, and these results are not generalizable to the asymmetric landings or cutting movements associated with a large number of ACL injuries. No conclusions can be made regarding injuries occurring by contact mechanisms.

Our study confirms findings by Ishida et al. (Ishida et al. 2015), who conducted a foot landing position study on female athletes and similarly observed increased knee abduction angles with toe-in landing and decreased knee abduction angles with toe-out landing. Our study additionally found decreases in knee flexion angle and moment in a toe-in landing position, whereas Ishida et al. did not, which may be attributed to methodological differences. We expanded on the study by Ishida and colleagues by including both males and females. Furthermore, Ishida et al. instructed subjects to land with maximal internal and external foot position allowed by comfort (range: -2.7 to 20.3°), whereas we asked subjects to land at pre-templated positions at 30° foot rotation from neutral, the magnitude considered “high-risk” in the Landing Error Scoring System (LESS) (Padua et al. 2009). Lastly, we evaluated a shod foot jump-landing task from a distance onto the force plates, whereas the former study evaluated barefoot drop-jump-landing activity.

## Conclusion

Landing in the toe-in position increases a number of biomechanical risk factors for ACL injury, including kinematic variables associated with a dynamic knee valgus position, and therefore should be avoided. The adverse biomechanical effects of toe-in landing position are exacerbated in females. Changing foot landing position appears to significantly alter lower extremity biomechanics for both men and women during a double-leg jump and can be a target for movement pattern modification in both sexes.
